# Is cognitive reserve associated with cognitive function across stroke severity? A longitudinal study among Chinese stroke patients

**DOI:** 10.3389/fnagi.2025.1652238

**Published:** 2025-11-20

**Authors:** Yinuo Ou, Yanrong Zhang, Yalin Lv, Silian Ding, Xiangjing Kong, Hanzhang Xu, Bei Wu, Hui Wei, Juan Li

**Affiliations:** 1 Department of Nursing, Huashan Hospital, Fudan University, Shanghai, China; 2 School of Nursing, Fudan University, Shanghai, China; 3 Shanghai Fourth People’s Hospital affiliated to Tongji University, Shanghai, China; 4 Air Force Hospital of Eastern Theater Command, Nanjing, China; 5 School of Nursing, Duke University, Durham, NC, United States; 6 School of Medicine, Duke University, Durham, NC, United States; 7 Rory Meyers College of Nursing, New York University, New York, NY, Unites States; 8 National Medical Center for Neurological Disorders, Shanghai, China

**Keywords:** cognitive reserve, post stroke cognitive impairment, stroke severity, acute ischemic stroke, longitudinal study

## Abstract

**Background:**

Cognitive decline is common after stroke. This study assessed the longitudinal associations between cognitive reserve and post-stroke cognitive function and tested whether these associations differ across patients’ stroke severity.

**Methods:**

A longitudinal survey was conducted among 371 patients with acute ischemic stroke from four stroke centers in China from 2022 to 2023. Eligible patients were recruited at acute stage and followed up at 3 and 6 months after onset. Cognitive reserve was assessed by Cognitive Reserve Index questionnaire within 7 days after stroke onset. Cognitive function was assessed by Montreal Cognitive Assessment, Changsha Version at each time point. Stroke severity was assessed using the National Institutes of Health Stroke Scale at admission. Linear mixed models were applied to assess associations between cognitive reserve and cognitive function across stroke severity.

**Results:**

Among stroke survivors, cognitive function improved over time after the onset (*β_*time*_* = 0.40, *p*<0.001). Higher level of cognitive reserve was associated with better cognitive function (*β_*cognitive reserve*_* = 0.08, *p*<0.001) after controlling for covariates. These associations remained over time (*β_*cognitive reserve*_ _*time*_* = 0.01, *p* = 0.009) and did not differ across patients with different stroke severity (*β_*cognitive reserve*_ _*stroke*_*_*severity*_ = 0.01, *p* = 0.11).

**Conclusion:**

Cognitive reserve can potentially mitigate the impact of stroke on long-term cognitive decline in Chinese patients with acute ischemic stroke. Targeting cognitive reserve may be a viable strategy to prevent or slow post-stroke cognitive decline. The study supports a more nuanced assessment of cognitive reserve as a standard in clinical studies.

## Introduction

Stroke is the leading cause of death and disability among Chinese adults, as well as the leading cause of disability-adjusted life year lost (Chunhua et al., 2023). It was estimated that there were 13 million stroke patients in China in 2022 (Mingbo et al., 2024). Among all stroke cases, acute ischemic stroke (AIS) was the most common type of stroke, accounting for 62.4% of all types (GBD 2017 Disease and Injury Incidence and Prevalence Collaborators, 2018). Stroke is associated with a variety of comorbidities, imposing substantial medical and economic burdens on both families and the society (Wang et al., 2022).

Post-stroke cognitive impairment (PSCI) has been observed in up to 38% of stroke survivors within 1 year after stroke (Sexton et al., 2019). It is defined as impairment of any of the six cognitive subgroups—visuoconstruction, attention, verbal memory, language, visual memory, and visuomotor function (Siow et al., 2024). PSCI is associated with reduced quality of life, increased stroke recurrence, and poorer functional outcomes in stroke patients (Claesson et al., 2005; Yu and Wang, 2024). With the increasing incidence and burden of stroke, PSCI has become a public health concern (Li et al., 2023). Therefore, it is critical to identify strategies to mitigate the risk of PSCI.

Reserve refers to the discordance between the degree of neuropathology and patients’ observed clinical manifestation (Stern, 2002). Cognitive reserve, an active subtype of reserve, is the brain’s ability to compensate for the pathological change in the brain by mobilizing neural networks, coping through preexisting cognitive processes, or by soliciting compensation processes (Stern, 2002; Stenberg et al., 2020; Dove et al., 2024). Specifically, cognitive reserve includes engaging in complex mental/lifestyle activities across the life span, such as educational attainment, professional achievement, and engagement in leisure and social (e.g., traveling and watching movies) activities (Stern, 2012). Previous studies have suggested that higher level of cognitive reserve could prevent the onset of disease-related clinical symptoms or the onset of age-related cognitive decline (Jia et al., 2022; Sánchez-Torres et al., 2023; Soldan et al., 2017). Similarly, in stroke patients, cognitive reserve can actively attempt to cope with or compensate for stroke pathology (Steffener et al., 2011; Steffener and Stern, 2012; Rosenich et al., 2020). Therefore, emerging evidence has suggested that cognitive reserve might be a modifiable factor that can buffer cognitive decline due to brain injury including stroke (Stern, 2002).

Still, most prior studies used static proxy indicators (e.g., years of education and/or occupational attainment) to measure cognitive reserve—a dynamic process—in stroke patients (Umarova et al., 2021; Shin et al., 2020; Umarova et al., 2019). In the present study, to address the limitation of static proxies, we used the Cognitive Reserve Index questionnaire (CRIq), a tool designed to integrate and quantify the cumulative contributions of education, occupation, and leisure activities across the entire life course (Nucci et al., 2012). To date, only two studies have comprehensively assessed the level of cognitive reserve among stroke survivors, and both showed that cognitive reserve had a protective effect on cognitive function post stroke (Abdullah et al., 2021; Li et al., 2022). However, both studies used a cross-sectional design, which were limited to assess the long-term trajectory of cognitive function in relation to cognitive reserve among stroke patients.

In this study, we first assessed whether cognitive reserve was associated with long-term cognitive function from stroke onset to 6 months in patients with AIS. We also tested whether the association vary across stroke severity.

## Materials and methods

### Participants

**FIGURE 1:**
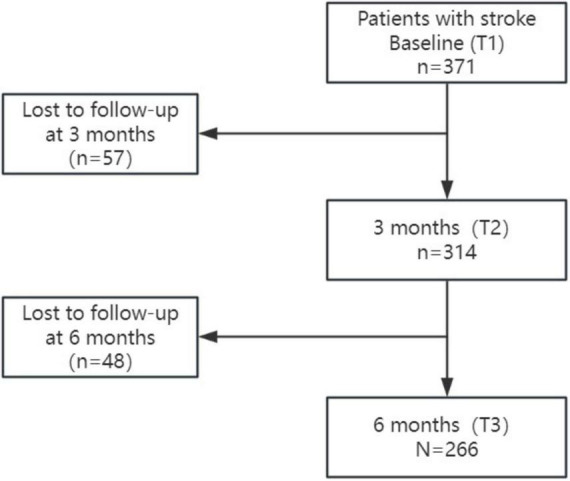
Flowchart of study participants.

We recruited AIS patients from four stroke centers in Shanghai and Nanjing, China, from September 2022 to December 2023. Patients were eligible if they were: (1) diagnosed with AIS by a neurologist or neurosurgeon based on focal neurological deficits and the corresponding cerebral magnetic resonance imaging infarction (MRI); (2) Aged ≥18 years; (3) admitted to stroke center within 7 days after stroke symptom onset. Exclusion criteria were: (1) having a medical history of mental illness or dementia prior to the stroke onset; (2) having a malignant disease with a life expectancy of less than 6 months; (3) having dysphasia or severe vision or hearing impairment or writing side upper limb paralysis. A total of 371 patients eligible for AIS were recruited in the study. Figure 1 presents the flowchart of enrolled participants and their follow-up status in this study.

### Procedures

One research assistant and one neurologist from each stroke center were trained using a standard study protocol. Research assistants, who typically held a bachelor’s or master’s degree in nursing, received specialized training on the MoCA. They all completed a MoCA administration certification program to ensure standardized and reliable application. Prior to the study’s commencement, all personnel also underwent comprehensive training on the overall study workflow, patient interaction, and data recording. The neurologists were responsible for assessing stroke severity. The research assistants collected baseline data from patients within 7 days of stroke onset face-to-face and assessed their cognitive reserve and cognitive function. At 3 and 6 months after the stroke onset, research assistants followed up with patients and assessed their cognitive function during in-person outpatient visits. This study complied with the Declaration of Helsinki and written informed consent was provided by all participants. The Institutional Review Board (IRB) of Huashan Hospital Fudan University approved the study (KY2022-829).

### Measurements

#### Cognitive reserve

We used the Cognitive Reserve Index questionnaire, an established instrument, to measure cognitive reserve that individuals have accumulated throughout their lifespan (Nucci et al., 2012). CRIq consists of three domains: (1) CRI-Education (CRI-E: The score is the sum of years in education and years in training courses throughout the lifespan); (2) CRI-Working Activity (CRI-WA: It is divided into five categories according to the level of cognitive commitment and responsibility required by the occupation; The total score is the product of each job level and years of work); (3) CRI-Leisure Time (CRI-LT: Measured by cognitively stimulating activities performed during leisure time throughout adulthood). The score of CRIq was then categorized into 5 levels: low (<70), moderate low (70–84), moderate (85–114), moderate high (115–130), and high ( ≥ 130). The Cronbach ’α of the Chinese version of CRIq is 0.839, and the retest reliability is 0.994 (Li and Li, 2022).

#### Cognitive function

We used the Chinese version of the Montreal Cognitive Assessment (MoCA), specifically the MoCA Changsha (MoCA-CS) (Tu et al., 2013), to assess patients’ cognitive function within 7 days after the stroke onset, at 3 months, and at 6 months. MoCA evaluates cognitive function across eight domains: Visuospatial, Executive, Naming, Attention, Language, Abstraction, Delayed recall and Orientation (Nasreddine et al., 2005). It has been widely used to assess cognitive function in stroke patients (Mao et al., 2020; Ozen et al., 2021). In our analysis, visuospatial and executive functions were combined into one domain as these two were assessed simultaneously using the clock drawing test (Kang et al., 2009; Pendlebury et al., 2012). The MoCA-CS has a score range of 0 to 30 points, with higher scores indicating better cognitive performance. For participants with fewer than 6 years of education, one point was added to the total score. The Cronbach’s α for the MoCA-CS was 0.884 (Tu et al., 2013).

#### Stroke severity

We evaluated patients’ stroke severity using the National Institutes of Health Stroke Scale (NIHSS) both at admission and before discharge. This 15-item neurologic exam takes less than 10 min to complete and has been widely used to measure stroke severity even in patients with language and cognitive impairments (Brott et al., 1989). The total score of the NIHSS has a range of 0 to 42, with higher scores indicating greater stroke severity (Spilker et al., 1997). The score of NIHSS was then categorized into 3 levels: NIHSS <5,mild stroke; NIHSS 5-13, moderate stroke; NIHSS >13, severe stroke (Schlegel et al., 2003).

### Covariates

All covariates were assessed at baseline and are listed in Supplementary Table 1. Patients’ sociodemographic characteristics were obtained from a survey questionnaire and included age, sex, ethnicity, marital status, rural/urban residency, medical insurance and years of education. Baseline clinical characteristics were extracted from the medical records that covered pain, hemiplegia, classification of stroke[the Oxfordshire Community Stroke Project (OCSP) classification and the Trial of ORG 10172 in Acute Stroke Treatment (TOAST) classification] (Adams et al., 1993; Lindley et al., 1993), comorbidities (e.g., hypertension, diabetes, dyslipidemia), and stroke risk factors (e.g., Body Mass Index, previous history of stroke, smoking status).

### Statistical analysis

Socio-demographic characteristics, clinical characteristics, stroke severity, cognitive reserve, and cognitive function were reported as median and interquartile range (IQR) for continuous variables and as frequency and proportion for categorical variables. We used full information maximum likelihood estimation within our linear mixed model to handle missing data from attrition. For true missing values, Little’s test supported a missing completely at random (MCAR) mechanism (*p* > 0.05). The scores of cognitive reserve (CRIq) and stroke severity (NIHSS) were treated as continuous variables in the analysis. We used the Kruskal–Wallis H test to compare continuous variables and the χ^2^ or Fisher’s exact test to compare categorical variables in assessing patients’ characteristics across the four centers and between patients lost to follow-up and those retained at each time point. We first estimated a series of general linear mixed-effect models to parameterize the longitudinal relationship between cognitive reserve and cognitive function overtime (Supplementary Table 2). We examined model assumptions including linearity, normality of residuals, and homoscedasticity using residual plots and Q–Q plots, and found no major violations. Specifically, we first estimated a base model by only adjusting for time (Models I and II). We then added cognitive reserve to the model and tested the interaction between cognitive reserve and time (Models III and IV). We used Akaike information criterion (AIC) and the Bayesian information criterion (BIC) to identify the best fitting model that describes the trajectory of cognitive decline over time (Supplementary Table 3). We then assessed the bivariate relationships between cognitive reserve, stroke severity, covariates and cognitive function. Only covariates that were significant (*P* < 0.1) in the univariate general linear mixed model and had weak correlation (*r* < 0. 2) with stroke severity (NIHSS at admission) and cognitive reserve (Supplementary Tables 2, 4) were entered into the multivariate linear mixed-effect models (Models V and VI). We then test the interaction between NIHSS at admission and cognitive reserve in the multivariate linear mixed models (Models VII). All models included a random intercept for each participant to capture individual-specific variability in baseline cognitive function. The center was not included as a random effect due to high collinearity with measured fixed-effect covariates (e.g., stroke severity and treatment methods for ischemic stroke), which more efficiently accounted for the inter-center variability. All analyses were conducted using IBM SPSS Statistics 27.0 (IBM Corp., Armonk, New York, USA).

**FIGURE 2:**
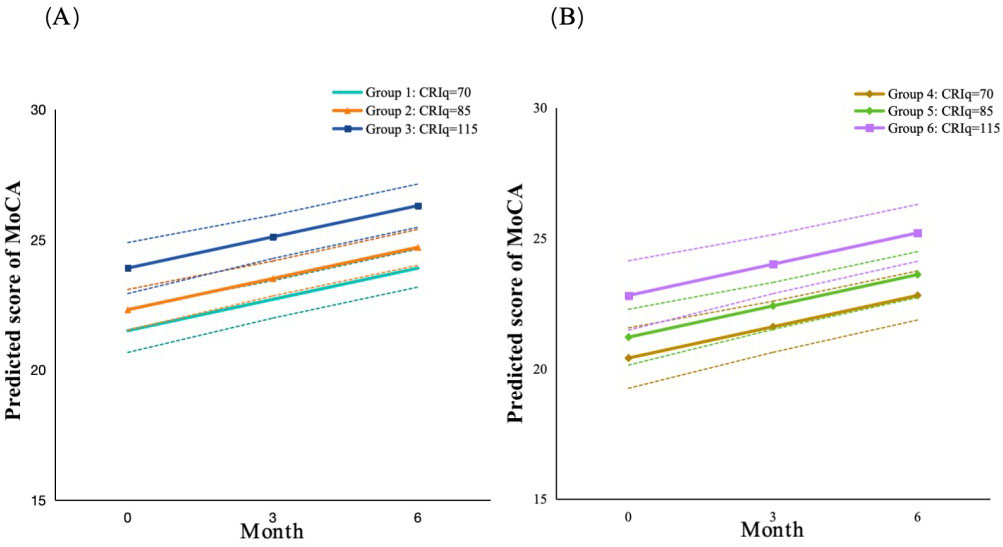
Trajectories of cognitive function (MoCA-CS) of AIS patients by stroke severity (NIHSS) and cognitive reserve (CRIq). Cognitive reserve was stratified into low (CRIq ≤ 84), moderate (85 ≤ CRIq ≤ 114), and high (CRIq ≥ 115). Stroke severity was categorized into mild (NIHSS < 5), moderate (NIHSS 5-13), and severe (NIHSS > 13). **(A)** Patients with mild stroke; **(B)** Patients with moderate stroke.

Based on a cross-classification of cognitive reserve and stroke severity, patients were stratified into distinct trajectory groups. This design resulted in six patient subgroups whose cognitive trajectories are illustrated in Figure 2.

## Results

Table 1 presents the baseline sociodemographic and clinical characteristics of patients. The median age of participants was 65 years (IQR:56–71) and median years of education was 9 years (IQR:9–12). Seventy-three-point-three percent were male. The median NIHSS at admission was 2 (IQR:1–3). The median cognitive reserve was 86 (IQR:80–98). The median score of MoCA-CS at baseline was 23 (IQR:18–27). The median score of MoCA-CS at baseline increased to 26 (IQR: 21–28) at 3-month and 26 (IQR: 23–28) at 6-month (Table 2). Comparison of baseline characteristics between participants who completed the 6-month follow-up and those lost to follow-up revealed significant differences in several variables. Participants lost to follow-up were more likely to have more severe functional impairment at discharge (higher mRs scores) and a higher proportion of hemiplegia. In addition, individuals without a history of previous stroke were more likely to be lost to follow-up compared to those with a history of stroke. Differences were also observed in TOAST classification, medical insurance coverage, and ethnicity. These differences are presented in detail in Supplementary Table 5.

**TABLE 1:** Baseline characteristics of patient samples from four stroke centers.

Characteristics		Center A (*n* = 50)	Center B (*n* = 117)	Center C (*n* = 104)	Center D (*n* = 100)	Pooled data (*N* = 371)	*P*-value
Age, years (IQR)	Han	66 (58.75–71.25)	59 (52–69)	66.5 (63–73)	63.5 (54–71)	65 (56–71)	<0.001
Ethnicity (%)		49 (98.0%)	117 (100.0%)	103 (99.0%)	100 (100.0%)	368 (99.2%)	0.24
Gender (%)	Female	14 (28.0%)	28 (23.9%)	30 (28.8%)	27 (27.0%)	99 (26.7%)	0.86
Marital status (%)	Currently married	42 (84.0%)	105 (89.7%)	91 (87.5%)	94 (94.0%)	332 (89.5%)	0.24
Residency (%)	Rural	10 (20.0%)	13 (11.1%)	16 (15.4%)	27 (27.0%)	66 (17.8%)	0.02
Medical insurance (%)	Basic insurance	41 (82%)	106 (90.6%)	95 (91.3%)	89 (89%)	331 (89.2%)	0.04
Education years (IQR)							0.12
	New rural co-operative medical system	4 (8%)	7 (6%)	8 (7.7%)	7 (7%)	26 (7.0%)	
	At one’s own expense	1 (2%)	4 (3.4%)	1 (1%)	4 (4%)	10 (2.7%)	
	Other	4 (8%)	0 (0%)	0 (0%)	0 (0%)	4 (1.1%)	
	0–2	9 (8–12)	10 (9–14.5)	12 (9–12)	9 (9–12)	9 (9–12)	
NIHSS at admission (IQR)		2 (1–4)	2 (0–3)	1 (1–2)	3 (2–4.75)	2 (1–3)	<0.001
NIHSS at discharge (IQR)		0 (0–2)	1 (0–2)	0 (0–1)	1 (0–2)	1 (0–2)	<0.001
mRs at discharge (%)		39 (78%)	109 (93.2%)	91 (87.5%)	73 (73%)	312 (84.1%)	<0.001
Hemiplegia (%)							<0.001
	3–6	11 (22%)	8 (6.8%)	13 (12.5%)	27 (27%)	59 (15.9%)	
	TACI	4 (8.0%)	24 (20.5%)	26 (25.0%)	38 (38.0%)	92 (24.8%)	
Pain (IQR)		0 (0–0)	0 (0–0)	0 (0–0)	0 (0–0)	0 (0–0)	<0.001
length of stay (IQR)		13 (11–14)	9 (7–11)	11 (9–13)	10 (7–12)	10 (8–13)	<0.001
OSCP classification (%)		0 (0%)	10 (8.5%)	3 (2.9%)	6 (6.0%)	19 (5.1%)	<0.001
TOAST classification (%)							<0.001
	PACI	14 (28.0%)	58 (49.6%)	66 (63.5%)	63 (63.0%)	201 (54.2%)	
	POCI	7 (14.0%)	27 (23.1%)	28 (26.9%)	25 (25.0%)	87 (23.5%)	
	LACI	29 (58.0%)	22 (18.8%)	7 (6.7%)	6 (6.0%)	64 (17.3%)	
	LAA	5 (10.0%)	43 (36.8%)	54 (51.9%)	27 (27.0%)	129 (34.8%)	
Comorbidities (%)							0.03
	CE	4 (8.0%)	3 (2.6%)	5 (4.8%)	7 (7.0%)	19 (5.1%)	
	SAO	21 (42.0%)	48 (41.0%)	45 (43.3%)	52 (52.0%)	166 (44.7%)	
	ODE	15 (30.0%)	11 (9.4%)	0 (0%)	7 (7.0%)	33 (8.9%)	
	UDE	5 (10.0%)	12 (10.3%)	0 (0%)	7 (7.0%)	24 (6.5%)	
	Hypertension	39 (78.0%)	78 (66.7%)	87 (83.7%)	72 (72.0%)	276 (74.4%)	
BMI (IQR)							
	Diabetes	24 (48.0%)	42 (35.9%)	28 (26.9%)	36 (36.0%)	130 (35.0%)	0.08
	Dyslipidemia	11 (22.0%)	30 (25.6%)	4 (3.8%)	31 (31.0%)	76 (20.5%)	<0.001
	Chronic obstructive pulmonary disease	0 (0%)	0 (0%)	2 (1.9%)	0 (0%)	2 (0.5%)	0.32
	Atherosclerosis	9 (18.0%)	0 (0%)	5 (4.8%)	1 (1.0%)	15 (4.0%)	<0.001
	Atrial fibrillation	3 (6.0%)	3 (2.6%)	6 (5.8%)	5 (5.0%)	17 (4.6%)	0.57
	Hyperuricemia	2 (4.0%)	0 (0%)	2 (1.9%)	0 (0%)	4 (1.1%)	0.04
	Coronary heart disease	3 (6.0%)	3 (2.6%)	7 (6.7%)	15 (15.0%)	28 (7.5%)	0.007
		24.20 (22.70–26.40)	25 (22.90–27.70)	24 (22.53–26.18)	24.80 (22.68–27.08)	24.50 (22.70–26.70)	0.28
Prior stroke (%)		17 (34.0%)	36 (30.8%)	36 (34.6%)	11 (11.0%)	100 (27%)	<0.001
Carotid atherosclerosis (%)		25 (50.0%)	85 (72.6%)	90 (86.5%)	78 (78.0%)	278 (74.9%)	<0.001
Smoking (%)		21 (42.0%)	23 (19.7%)	46 (44.2%)	47 (47.0%)	137 (36.9%)	<0.001
MoCA-CS (IQR)	Visuospatial /executive	3 (2.75–5)	3 (1–4)	3 (1–4)	2 (0–4)	3 (1–4)	0.002
	Naming	3 (3–3)	3 (2–3)	3 (3–3)	3 (2–3)	3 (3–3)	<0.001
	Attention	5 (3–6)	6 (5–6)	6 (5–6)	5 (4–6)	5 (4–6)	0.004
	Language	2 (2–3)	2 (1.50–3)	2 (2–3)	1 (1–2)	2 (1–3)	<0.001
	Abstraction	2 (2–2)	2 (1–2)	2 (1–2)	1 (0–1)	2 (1–2)	<0.001
	Delayed recall	3 (1.75–4)	3 (1–3)	3 (1–4)	2 (0–3)	2 (1–4)	0.15
	Orientation	6 (5–6)	6 (5–6)	6 (6–6)	5 (4–6)	6 (5–6)	<0.001
	Total	24 (20.75–27)	23 (19–27)	25 (20–27.75)	21 (15–24)	23 (18–27)	<0.001
Treatment methods for ischemic stroke (%) Cognitive reserve (IQR)	Non-guideline recommended treatment	43 (86.0%)	114 (97.4%)	78 (75.0%)	66 (66.0%)	301 (81.1%)	<0.001 0.29
	Guideline recommended treatment*^a^*	7 (14.0%)	3 (2.6%)	26 (25.0%)	34 (34.0%)	70 (18.9%)	
	CRIq	89.50 (80.75–106.25)	86 (78–98)	86 (81–96.75)	84.5 (80–95)	86 (80–98)	
	CRI-education	101 (91.5–115)	97 (89.5–108)	102.50 (96–108)	98 (90.25–104)	99 (92–108)	0.02
	CRI-occupation	95 (86.75–123.25)	94 (86–108)	90 (85–107)	91.50 (86–104.75)	92 (86–108)	0.27
	CRI-leisure activity engagement	78 (71.75–85)	76 (68.50–85)	76 (70–82)	77 (71.25–83)	77 (70–83)	0.36

BMI, Body mass index; CRIq, Cognitive Reserve Index questionnaire; CE, cardioembolic; IQR, interquartile range; LACI, lacunar circulation infarcts; LAA, large-artery atherothrombotic; MoCA-CS, Montreal Cognitive Assessment-Changsha Version; mRs, Modified Rankin Scale; NIHSS, National Institutes of Health Stroke Scale; OCSP, Oxfordshire Community Stroke Project; ODE, other determined etiology; POCI, posterior circulation infarct; PACI, partial anterior circulation infarct; SAO, small-artery occlusion; TACI, total anterior circulation infarct; TOAST, Trial of ORG 10172 in Acute Stroke Treatment; UDE, undetermined etiology.

*^a^*Guideline recommended treatments included intravenous thrombolysis, intra-arterial thrombectomy, endovascular stent implantation, and balloon dilation.

**TABLE 2:** Cognitive function at acute stage, 3rd month and 6th month.

Variables	Acute stage	3rd month	6th month	*B* (95%*CI*)	*P*
Visuospatial /executive	3 (1–4)	4 (2–5)	4 (3–5)	0.16 (0.11,0.20)	<0.001
Naming	3 (3–3)	3 (3–3)	3 (3–3)	0.02 (0.004,0.04)	0.02
Attention	5 (4–6)	6 (5–6)	6 (5–6)	0.06 (0.03,0.10)	<0.001
Language	2 (1–3)	2 (2–3)	3 (1–3)	0.04 (0.01,0.06)	<0.001
Abstraction	2 (1–2)	2 (1–2)	2 (1–2)	0.03 (0.01,0.05)	0.01
Delayed recall	2 (1–4)	3 (2–4)	3 (2–4)	0.10 (0.06,0.14)	<0.001
Orientation	6 (5–6)	6 (6–6)	6 (6–6)	0.05 (0.02,0.08)	<0.001
MoCA-CS	23 (18–27)	26 (21–28)	26 (23–28)	0.45 (0.30,0.60)	<0.001

CI, confidence interval; MoCA-CS, Montreal Cognitive Assessment-Changsha Version.

Table 3 presents the results from multivariate linear mixed models of cognitive reserve and stroke severity on cognitive function. We found that scores of MoCA-CS increased over time after controlling for covariates (β = 0.40, *P* < 0.001), indicating that scores of MoCA-CS increased by 0.40 point per month. Cognitive reserve was significantly and positively associated with cognitive function. Participants with higher level of cognitive reserve showed better cognitive function (β = 0.08, *P* < 0.001). Stroke severity was negatively associated with cognitive function. Participants who with higher level of stroke severity had lower scores in MoCA-CS (β = −0.22, *P* < 0.001). The interaction between cognitive reserve (CRIq) and stroke severity (NIHSS) was not significant (β = 0.01, *P* = 0.11), which indicated that cognitive reserve was a protective factor for longitudinal cognitive function after stroke regardless stroke severity. Figure 2 demonstrated the trajectories of cognitive function by cognitive reserve and stroke severity after controlling for other covariates. Visual inspection of the faceted plot suggests that the influence of cognitive reserve on MoCA-CS scores is more pronounced than that of stroke severity. Specifically, within each level of stroke severity (mild or moderate), the differences in trajectories between cognitive reserve groups are evident and consistent over time.

**TABLE 3:** The results from multivariate linear mixed models on the effect of cognitive reserve on stroke severity and cognitive function.

Variables	Model V	Model VI	Model VII
Fixed-effects parameters			
Intercept	14.80** (11.55, 18.06)	17.43** (12.42, 22.43)	19.90** (14.03, 25.76)
Time	0.40** (0.30, 0.50)	0.40** (0.30, 0.50)	0.40** (0.30, 0.50)
CRIq	0.09** (0.05, 0.12)	0.08** (0.05, 0.12)	0.05* (0.003, 0.10)
NIHSS	−0.27** (−0.44, −0.11)	−0.22** (−0.40, −0.05)	−1.19 (−2.41, 0.03)
CRIq*NIHSS	13.97** (12.44, 15.69)	−0.04 (−0.09, 0.000)	0.01 (−0.002, 0.03)
Age			−0.04 (−0.08, 0.004)
Diabetes		−0.97 (−1.99, 0.05)	−1.05* (−2.07, −0.03)
Treatment methods for ischemic stroke		0.10 (−1.16, 1.37)	0.20 (−1.07, 1.47)
Non-guideline recommended treatment (reference)			
Guideline recommended treatment*^a^*			
OSCP		1.17 (−1.14, 3.48)	0.91 (−1.42, 3.24)
TACI (reference)			
PACI			
POCI		2.00 (−0.42, 4.42)	1.68 (−0.77, 4.13)
LACI		2.17 (−0.36, 4.71)	1.91 (−0.64, 4.46)
TOAST			
LAA (reference)			
CE		−1.02 (−3.32, 1.29)	−0.96 (−3.26, 1.34)
SAO		0.21 (−0.90, 1.32)	0.25 (−0.87, 1.36)
ODE		0.06 (−1.79, 1.90)	0.11 (−1.73, 1.95)
UDE		−0.83 (−2.87, 1.22)	−0.72 (−2.77, 1.32)
Residency		−1.21 (−2.50, 0.07)	−1.18 (−2.46, 0.11)
Carotid atherosclerosis		−0.45 (−1.63, 0.73)	−0.44 (−1.62, 0.74)
Random-effects variance		14.01** (12.48, 15.74)	14.01** (12.47, 15.74)
Level 1: Within-person			
Level 2: Intercept	15.73** (12.80, 19.33)	15.19** (12.27, 18.80)	15.11** (12.20, 18.71)
Goodness-of-Fit	5715.55	5689.25	5694.81
AIC value			
BIC value	5725.26	5698.93	5704.49

AIC, Akaike information criterion; BIC, Bayesian information criterion; CRIq, Cognitive Reserve Index questionnaire; CI, confidence interval; CE, cardioembolic; LAA, large-artery atherothrombotic; NIHSS, National Institutes of Health Stroke Scale (at admission); ODE, other determined etiology; SAO, small-artery occlusion; UDE, undetermined etiology. Estimated coefficients (95% confidence intervals) are reported.

*^a^*Guideline recommended treatments included intravenous thrombolysis, intra-arterial thrombectomy, endovascular stent implantation, and balloon dilation.

*p < 0.05,

**p < 0.001.

## Discussion

In this study, we investigated the trajectory of cognitive function in Chinese AIS patients over 6 months and examined the role of cognitive reserve on cognitive function across stroke severity. We found that cognitive function improved in stroke patients over time. Cognitive reserve was positively associated with cognitive function across stroke severity after controlling for key covariates such as age, education, diabetes, treatment methods for ischemic stroke, OSCP, TOAST, residency and carotid atherosclerosis. Using a more comprehensive measure of cognitive reserve, a key finding of our study is the potentially stronger protective role of cognitive reserve compared to the initial impact of stroke severity on long-term cognitive outcomes. These findings provided new evidence that cognitive reserve played protective role in long-term cognitive function after stroke. Our findings support the protective role of cognitive reserve in preserving cognitive function and sustaining recovery among stroke survivors. In addition, we found that etiological classification of ischemic stroke was not associated with post-stroke cognitive impairment, which is consistent with previous research (Li et al., 2022).

The results of this study are consistent with previous longitudinal studies using cognitive reserve proxies, demonstrating that higher cognitive reserve is associated with better cognitive function within 6 months after stroke (Ding et al., 2019; Dong et al., 2021; Shin et al., 2020). These results suggest that cognitive reserve can be used as an independent predictor of cognitive performance after stroke. The cognitive reserve model assumes that cognitive reserve mainly compensates for pathological cognitive impairment through two mechanisms: neural capacity and neural efficiency (Cabeza et al., 2018). Neural capacity refers to the neural resources available for cognition, such as stimulating activities such as education, work, and recreation. Neural efficiency refers to the ability to utilize these neural resources by using less neural resources, which is often operationalized as neural activity, to perform a cognitive task. Patients with high levels of cognitive reserve appear to better withstand more severe stroke-related pathology compared to those with lower reserve, thereby delaying the decline of cognitive function (Stern, 2009). Our findings support the hypothesis of the cognitive reserve model and highlight the protective effect of cognitive reserve on cognitive function.

Early research had hypothesized that cognitive reserve moderated the relationship between brain pathology and clinical outcomes among stroke patients (Rosenich et al., 2020). Previously, our team found the moderating effect of cognitive reserve on the relationship between stroke severity and cognitive function based on cross-sectional data (Li et al., 2022). However, such moderating effect was not observed in the present longitudinal analysis. One reason may be the measurement of brain pathology. While previous research suggested using neuroimaging data such as stroke volume and location to assess brain pathology, we applied NIHSS, a valid and widely used stroke severity scale, to assess stroke-related brain pathology rather than neuroimaging data. The differences between stroke volume, location and clinical manifestations may impact the outcomes. Future longitudinal studies using neuroimage data to indicate stroke brain pathology are needed to explore the moderating effect of cognitive reserve between stroke brain pathology and long-term patients’ outcomes. Another reason may be the measurement of cognitive reserve. Cognitive reserve was a theoretical concept, usually measured by indicators. Previous research used education and/or occupation and/or age as proxies of cognitive reserve (Umarova et al., 2021; Umarova et al., 2019; Shin et al., 2020), however, our study used CRIq to comprehensively assess and measure the amount of cognitive reserve accumulated by individuals over their lifetime including education and years in training courses throughout the lifespan, working activity and leisure time. The differences between different measures of cognitive reserve may impact the outcomes.

Cognitive reserve is an ability of the brain to actively buffer the damage caused by neuropathology, which can be used to explain the heterogeneity of the trajectory of cognitive function change in different individuals (Stern, 2012). Among individuals with the same level of brain structural capacity, those with higher cognitive reserve are more likely to withstand more severe brain pathological damage and show better cognitive ability than individuals with lower reserve (Cui et al., 2024). Cognitive reserve is plastic and will continue to develop and accumulate over the course of an individual’s life. Early-life cognitive ability, established by young adulthood, is a fundamental component of cognitive reserve and a powerful predictor of later-life cognitive function (Kremen et al., 2022; Schwarz et al., 2024). According to the cognitive reserve theory, educational attainment is a key cognitive stimulus that strengthens cognitive reserve before adulthood. Furthermore, individual involvement in cognitive-related occupations in adulthood enhanced the ability of the cognitive reserve-shaped brain to compensate for pathological damage through neural compensation, which buffered cognitive decline in older age (Stern, 2002). Higher levels of employment are more mentally challenging than less complex jobs, generating additional neuronal resources that can prevent cognitive decline (Scarmeas and Stern, 2003; Xue et al., 2018). However, as these factors are largely static in later life, they cannot fully capture the dynamic, ongoing nature of cognitive reserve accumulation. This underscores the importance of modifiable factors, such as engagement in leisure activities, which are associated with promoted neuroplasticity and protection against cognitive decline even in old age (Wang et al., 2012). It is plausible that this relationship reflects a protective effect, or that individuals with higher cognitive reserve are more likely to engage in such activities. To address the limitation of static proxies, we used the CRIq, a tool designed to integrate and quantify the cumulative contributions of education, occupation, and leisure activities across the entire life course. Enhancing leisure activities at all stages of an individual’s life course is an important strategy to preserve cognitive function after stroke.

This study also had several limitations: (1) The stroke severity of participants in this study was mild to moderate, so we were unable to examine the role of cognitive reserve in cognitive function among survivors of more severe strokes. (2) Only three time points were available: baseline, 3 months and 6 months. The change of cognitive function is a long-term process, and future research should continue to explore the role of cognitive reserve on long-term cognitive function. (3) We did not collect information on infarct locations, focal volume and cognitive training, which may be related to cognitive function after stroke (Mijajlović et al., 2017). (4) The percentage of participants lost to follow-up was relatively high. These individuals generally had more severe stroke impairment, lower cognitive function, and more unfavorable functional outcomes at baseline. This may lead to an overestimation of cognitive function outcomes. (5) We utilized the NIHSS to quantify stroke severity. It is known to be weighted more heavily toward language deficits, which are often associated with left hemisphere lesions, thereby tending to overestimate severity in left-sided strokes and underestimate right-sided strokes (Woo et al., 1999). This systematic asymmetry could influence the interpretation of our findings regarding the relationship between baseline stroke severity and cognitive outcomes. Future studies would benefit from incorporating additional complementary measures to provide a more balanced and comprehensive evaluation of stroke severity across both hemispheres.

## Conclusion

This longitudinal study supports a positive role of cognitive reserve in promoting long-term cognitive function after stroke among Chinese AIS patients. Critically, in patients with mild to moderate stroke-related pathological burden, our findings demonstrate that cognitive reserve plays a protective role in preserving long-term cognitive function. This indicates that higher levels of cognitive reserve are associated with maintained cognitive performance over time. Cognitive reserve represents a promising target for strategies aimed at preventing or mitigating cognitive decline after stroke. The study supports a more nuanced assessment of cognitive reserve as a standard in clinical studies.

## Data Availability

The raw data supporting the conclusions of this article will be made available by the authors, without undue reservation.
