# Supplementary material for: Is cognitive reserve associated with cognitive function across stroke severity? A longitudinal study among Chinese stroke patients
Source: Front Aging Neurosci. 2025 Nov 20;17:1652238. doi: 10.3389/fnagi.2025.1652238 (PMC12676486; doi:10.3389/fnagi.2025.1652238)
Supplement: Supplementary file 1 [file Table_1.docx]

**Supplementary Table S1** The list of covariates.

| Covariates | Variable |  | Coding |
| --- | --- | --- | --- |
| Sociodemographic characteristics | Age | Continuous variable |  |
|  | Ethnicity | Categorical variable | Han=1, Non-Han=0 |
|  | Sex | Categorical variable | Female=1, man=0 |
|  | Marital status | Categorical variable | Currently married=1  other=0 |
|  | Residency | Categorical variable | Rural=1, other=0 |
|  | Education years | Continuous variable |  |
| Severity of stroke | NIHSS at admission | Continuous variable |  |
|  | NIHSS at discharge | Continuous variable |  |
|  | Hemiplegia | Categorical variable | Yes=1, No=0 |
|  | Pain | Continuous variable |  |
|  | Modified Rankin Scale at discharge | Categorical variable | 0-2=0, 3-6=1 |
| Classification of stroke | Oxfordshire Community Stroke Project (OSCP)  Classification | Categorical variable | LACI=1  PACI=2  POCI=3  TACI=4 |
|  | Trial of ORG 10,172 in Acute Stroke Treatment  (TOAST) classification | Categorical variable | UDE=1  CE=2  SAO=3  ODE=4  LAA=5 |
| Comorbidities | Hypertension | Categorical variable | Yes=1, No=0 |
|  | Diabetes | Categorical variable | Yes=1, No=0 |
|  | Dyslipidemia | Categorical variable | Yes=1, No=0 |
|  | Chronic obstructive pulmonary disease | Categorical variable | Yes=1, No=0 |
|  | Atherosclerosis | Categorical variable | Yes=1, No=0 |
|  | Atrial fibrillation | Categorical variable | Yes=1, No=0 |
|  | Hyperuricemia | Categorical variable | Yes=1, No=0 |
|  | Coronary heart disease | Categorical variable | Yes=1, No=0 |
|  | Others | Categorical variable | Yes=1, No=0 |
| Stroke risk factors | Body mass index (BMI) | Continuous variable |  |
|  | Previous history of stroke | Categorical variable | Yes=1, No=0 |
|  | Carotid atherosclerosis | Categorical variable | Yes=1, No=0 |
|  | Smoking | Categorical variable | Yes=1, No=0 |
|  | Length of stay | Continuous variable |  |
| Others | Treatment methods for ischemic stroke | Categorical variable | Non-guideline recommended treatment=0  Guideline recommended treatment^a^=1 |
|  | Stroke center | Categorical variable | Center A=1  Center B=2  Center C=3  Center D=4 |
|  | Medical insurance | Categorical variable | Basic insurance =1  New Rural Co-operative Medical System=2  At one's own expense=3  Other=4 |

^a^ Guideline recommended treatments included intravenous thrombolysis, intra-arterial thrombectomy, endovascular stent implantation, and balloon dilation.

**Supplementary Table S2** Associations between participant characteristics and cognitive function using general linear mixed-effect models (N =371).

| Variables | | Coefficient (95%*CI*) | *P* |
| --- | --- | --- | --- |
|  |  |  |  |
| Cognitive reserve |  | 0.09 (0.06, 0.11) | ＜0.01 |
| Age (years) |  | -0.03 (-0.07, -0.01) | 0.04 |
| Han ethnicity |  | 0.81 (-3.69, 5.31) | 0.72 |
| Female |  | -0.17 (-0.97, 0.64) | 0.69 |
| Marital status (currently married) |  | -0.63 (-1.77, 0.51) | 0.28 |
| Rural residents |  | -2.19 (-3.11, -1.26) | ＜0.01 |
| Education in years |  | 0.28 (0.18, 0.37) | ＜0.01 |
| Length of stay |  | -0.02 (-0.11, 0.08) | 0.69 |
| NIHSS at admission |  | -0.31 (-0.43, -0.18) | ＜0.01 |
| NIHSS at discharge |  | -0.22 (-0.40, -0.04) | 0.02 |
| Pain |  | -0.14 (-0.92, 0.63) | 0.72 |
| Hemiplegia |  | -2.08 (-2.93, -1.23) | ＜0.01 |
| OSCP classification |  |  |  |
|  | TACI (reference) |  |  |
|  | PACI | 2.32 (0.69, 3.95) | 0.01 |
|  | POCI | 3.01 (1.28, 4.73) | ＜0.01 |
|  | LACI | 3.42 (1.66, 5.17) | ＜0.01 |
| TOAST classification |  |  |  |
|  | LAA (reference) |  |  |
|  | CE | -1.08 (-2.76, 0.61) | 0.21 |
|  | SAO | 0.59 (-0.23, 1.40) | 0.16 |
|  | ODE | 1.34 (0.06, 2.62) | 0.04 |
|  | UDE | -0.65 (-2.12, 0.82) | 0.39 |
| Hypertension |  | -0.19 (-1.00, 0.63) | 0.66 |
| Diabetes |  | -0.80 (-1.55, -0.05) | 0.04 |
| Dyslipidemia |  | 0.14 (-0.75, 1.04) | 0.75 |
| Chronic obstructive pulmonary disease |  | 3.13 (-2.50, 8.77) | 0.28 |
| Atherosclerosis |  | 0.37 (-1.42, 2.15) | 0.69 |
| Atrial fibrillation |  | 0.08 (-1.65, 1.81) | 0.93 |
| Hyperuricemia |  | 1.59 (-2.15, 5.32) | 0.41 |
| Coronary heart disease |  | -0.73 (-2.14, 0.67) | 0.31 |
| BMI |  | -0.08 (-0.19, 0.03) | 0.15 |
| Prior stroke |  | 0.48 (-0.32, 1.27) | 0.24 |
| Carotid atherosclerosis |  | -1.26 (-2.08, -0.44) | ＜0.01 |
| Smoking |  | 0.49 (-0.26, 1.23) | 0.20 |
| Treatment methods for ischemic stroke |  |  |  |
|  | Non-guideline recommended treatment (reference) |  |  |
|  | Guideline recommended treatment^a^ | -0.97 (0.48, -1.91) | 0.04 |
| Stroke center |  |  |  |
|  | Center A (reference) |  |  |
|  | Center B | -0.63 (0.53, -1.66) | 0.24 |
|  | Center C | 0.88 (0.55, -0.20) | 0.11 |
|  | Center D | -3.68 (0.58, -4.82) | ＜0.01 |
| mRs (at discharge) |  |  |  |
|  | 0-2 (reference) |  |  |
|  | 3-6 | -2.14 (-3.18, -1.11) | ＜0.01 |
| Medical insurance |  |  |  |
|  | Basic insurance (reference) |  |  |
|  | New Rural Co-operative Medical System | -3.48 (-4.82, -2.14) | ＜0.01 |
|  | No insurance | -0.81 (-3.13, 1.51) | 0.49 |
|  | Others | 3.31 (0.17, 6.45) | 0.04 |

Abbreviations: BMI, Body mass index; CRIq, Cognitive Reserve Index questionnaire; CE, cardioembolic; LACI, lacunar circulation infarcts; LAA, large-artery atherothrombotic; mRs, Modified Rankin Scale; NIHSS, National Institutes of Health Stroke Scale; OCSP, Oxfordshire Community Stroke Project; ODE, other determined etiology; PACI, partial anterior circulation infarct; POCI, posterior circulation infarct; SAO, small-artery occlusion; TACI, total anterior circulation infarct; TOAST, Trial of ORG 10172 in Acute Stroke Treatment; UDE, undetermined etiology.

^a^ Guideline recommended treatments included intravenous thrombolysis, intra-arterial thrombectomy, endovascular stent implantation, and balloon dilation.

**Supplementary Table S3** Model Building for Trajectories of Cognitive Reserve Over Time in Stroke Patients.

|  | Model Ⅰ | Model Ⅱ | Model Ⅲ | Model Ⅳ |
| --- | --- | --- | --- | --- |
| **Fixed-Effects Parameters** |  |  |  |  |
| Intercept | 22.00*** (21.44, 22.55) | 22.32*** (21.79, 22.86) | 13.60*** (10.37, 16.82) | 11.32*** (7.68, 14.96) |
| Time | 0.40*** (0.30, 0.50) |  | 0.40*** (0.30, 0.50) | 1.26*** (0.61, 1.92) |
| Time^2^ |  | 0.06*** (0.04, 0.07) |  |  |
| CRIq |  |  | 0.09*** (0.06, 0.13) | 0.12*** (0.08, 0.16) |
| Time*CRIq |  |  |  | 0.01** (0.002, 0.02) |
| **Random-Effects Variance** |  |  |  |  |
| Level 1: Within-person | 13.98*** (12.45, 15.70) | 14.36*** (12.79, 16.13) | 13.95*** (12.43, 15.67) | 13.86*** (12.34, 15.57) |
| Level 2: Intercept | 17.82*** (14.61, 21.75) | 17.77*** (14.54, 21.71) | 16.34*** (13.34, 20.02) | 16.27*** (13.28, 19.92) |
| **Goodness-of-Fit** |  |  |  |  |
| AIC value | 5743.09 | 5763.85 | 5723.04 | 5725.62 |
| BIC value | 5752.80 | 5773.56 | 5732.75 | 5735.33 |

Abbreviations: AIC, Akaike Information Criterion; BIC, Bayesian Information Criterion; CRIq, Cognitive Reserve Index questionnaire.

Note: Estimated coefficients (95% confidence intervals) are reported. Time and Time^2^ indicate linear and quadratic slope parameters, respectively.

* *p* < 0.05, ** *p* < 0.01, *** *p* < 0.001.

**Supplementary Table S4** Spearman's correlations between MoCA, and covariates that were significant in univariate general linear mixed model.

| Variables | Age | CRIq | NIHSS at admission | MoCA | Center B | Center C | Center D | Education years | NIHSS at discharge | Hemiplegia | Diabetes | Treatment methods for ischemic stroke | PACI | POCI | LACI | CE | SAO | ODE | UDE | Residency | Carotid atherosclerosis | Medical insurance | mRs |
| --- | --- | --- | --- | --- | --- | --- | --- | --- | --- | --- | --- | --- | --- | --- | --- | --- | --- | --- | --- | --- | --- | --- | --- |
| Age | — | 0.01 | -0.08 | -0.05 | -0.21** | 0.22** | -0.05 | -0.07 | -0.14** | -0.07 | 0.11* | 0.00 | 0.02 | 0.02 | 0.02 | 0.08 | 0.01 | -0.08 | -0.02 | -0.16** | 0.12* | -0.17** | 0.01 |
| CRIq | — | — | -0.07 | 0.29** | -0.03 | 0.01 | -0.05 | 0.42** | 0.00 | -0.13* | 0.03 | -0.11* | -0.05 | 0.02 | 0.07 | 0.06 | -0.04 | 0.11* | -0.04 | -0.14** | -0.08 | -0.14** | -0.06 |
| NIHSS at admission | — | — | — | -0.27** | -0.09 | -0.19** | 0.23** | -0.05 | 0.65** | 0.43** | 0.13* | 0.21** | -0.02 | -0.02 | -0.05 | 0.01 | 0.01 | -0.03 | -0.01 | 0.14** | 0.06 | 0.07 | 0.42** |
| MoCA | — | — | — | — | 0.02 | 0.15** | -0.25** | 0.24** | -0.21** | -0.23** | -0.09 | -0.10 | -0.07 | 0.04 | 0.14** | -0.02 | 0.03 | 0.03 | -0.04 | -0.11* | -0.14** | -0.04 | -0.18** |
| Center B | — | — | — | — | — | -0.42** | -0.41** | 0.05 | 0.07 | -0.07 | 0.01 | -0.28** | -0.06 | -0.01 | 0.03 | -0.08 | -0.05 | 0.01 | 0.11* | -0.12* | -0.04 | -0.03 | -0.17** |
| Center C | — | — | — | — | — | — | -0.38** | 0.08 | -0.30** | 0.00 | -0.11* | 0.10 | 0.12* | 0.05 | -0.17** | -0.01 | -0.02 | -0.20** | -0.16** | -0.04 | 0.17** | -0.05 | -0.06 |
| Center D | — | — | — | — | — | — | — | -0.06 | 0.24** | 0.19** | 0.01 | 0.24** | -0.11* | 0.02 | -0.18** | 0.05 | 0.09 | -0.04 | 0.01 | 0.15** | 0.04 | 0.00 | 0.18** |
| Education years | — | — | — | — | — | — | — | — | 0.01 | 0.03 | -0.01 | -0.04 | 0.04 | 0.01 | 0.01 | 0.01 | 0.03 | -0.02 | -0.03 | -0.37** | -0.05 | -0.21** | -0.02 |
| NIHSS at discharge | — | — | — | — | — | — | — | — | — | 0.38** | 0.12* | 0.06 | -0.06 | -0.01 | 0.00 | -0.04 | 0.00 | -0.03 | 0.06 | 0.09 | 0.03 | 0.03 | 0.42** |
| Hemiplegia | — | — | — | — | — | — | — | — | — | — | -0.07 | 0.03 | 0.14** | -0.07 | -0.10 | -0.11* | 0.09 | -0.14** | -0.08 | 0.06 | 0.03 | 0.06 | 0.37** |
| Diabetes | — | — | — | — | — | — | — | — | — | — | — | -0.04 | 0.01 | -0.10 | 0.10 | -0.04 | 0.08 | -0.03 | 0.01 | -0.11* | 0.06 | -0.13* | 0.10 |
| Treatment methods for ischemic stroke | — | — | — | — | — | — | — | — | — | — | — | — | 0.03 | -0.06 | -0.07 | 0.04 | -0.07 | -0.03 | -0.04 | 0.05 | 0.10* | -0.06 | 0.07 |
| PACI | — | — | — | — | — | — | — | — | — | — | — | — | — | -0.60** | -0.50** | -0.06 | 0.01 | -0.15** | -0.11* | -0.03 | 0.12* | -0.09 | 0.05 |
| POCI | — | — | — | — | — | — | — | — | — | — | — | — | — | — | -0.25** | -0.01 | -0.03 | 0.05 | -0.04 | 0.08 | 0.04 | 0.05 | -0.03 |
| LACI | — | — | — | — | — | — | — | — | — | — | — | — | — | — | — | 0.06 | 0.09 | 0.13* | 0.11* | -0.08 | -0.25** | -0.01 | -0.10 |
| CE | — | — | — | — | — | — | — | — | — | — | — | — | — | — | — | — | -0.21** | -0.07 | -0.06 | 0.12* | -0.12* | 0.11* | -0.03 |
| SAO | — | — | — | — | — | — | — | — | — | — | — | — | — | — | — | — | — | -0.28** | -0.24** | -0.04 | -0.11* | -0.02 | -0.05 |
| ODE | — | — | — | — | — | — | — | — | — | — | — | — | — | — | — | — | — | — | -0.08 | -0.02 | -0.19** | 0.02 | 0.02 |
| UDE | — | — | — | — | — | — | — | — | — | — | — | — | — | — | — | — | — | — | — | -0.01 | 0.00 | -0.05 | -0.02 |
| Residency | — | — | — | — | — | — | — | — | — | — | — | — | — | — | — | — | — | — | — | — | 0.01 | 0.53** | 0.11* |
| Carotid atherosclerosis | — | — | — | — | — | — | — | — | — | — | — | — | — | — | — | — | — | — | — | — | — | -0.07 | 0.05 |
| Medical insurance | — | — | — | — | — | — | — | — | — | — | — | — | — | — | — | — | — | — | — | — | — | — | 0.04 |

Abbreviations: CE, cardioembolic; CRIq, Cognitive Reserve Index questionnaire; LACI, lacunar circulation infarcts;mRs, Modified Rankin Scale; MoCA, Montreal Cognitive Assessment; NIHSS, National Institutes of Health Stroke Scale; ODE, other determined etiology; PACI, partial anterior circulation infarct; POCI, posterior circulation infarct; SAO, small-artery occlusion; UDE, undetermined etiology.

*Correlation is significant at p＜0.05 level.

**Correlation is significant at p＜0.01 level.

**Supplementary Table S5 Comparison of Baseline Characteristics Between Participants Retained and Those Lost to Follow-up at Each Time Point.**

| Characteristics | | 3-Month Follow-up | | | 6-Month Follow-up | | |
| --- | --- | --- | --- | --- | --- | --- | --- |
|  |  | Retained (n=314) | Lost (n=57) | p-value | Retained (n=266) | Lost (n=105) | p-value |
| Age，years (IQR) |  | 64 (56-70) | 65 (60-73) | 0.55 | 64 (56-70) | 65 (57-71) | 0.23 |
| Ethnicity (%) | Han | 312 (99.4%) | 57 (100%) | 0.55 | 264 (99.2%) | 105 (100%) | ＜0.001 |
| Gender (%) | Female | 85 (27.1%) | 14 (24.6%) | 0.69 | 74 (27.8%) | 25 (23.8%) | 0.43 |
| Marital status (%) | Currently married | 280 (89.2%) | 52 (91.2%) | 0.64 | 234 (88.0%) | 98 (93.3%) | 0.13 |
| Residency (%) | Rural | 58 (18.5%) | 8 (14.0%) | 0.42 | 46 (17.3%) | 20 (19.0%) | 0.69 |
| Medical insurance (%) |  |  |  | 0.57 |  |  | ＜0.001 |
|  | Basic insurance | 278 (88.5%) | 53 (93.0%) |  | 236 (88.7%) | 95 (90.5%) |  |
|  | New Rural Co-operative Medical System | 24 (7.6%) | 2 (5.3%) |  | 21 (7.9%) | 5 (4.8%) |  |
|  | At one's own expense | 8 (2.5%) | 2 (5.3%) |  | 5 (1.9%) | 5 (4.8%) |  |
|  | Other | 4 (1.3%) | 0 (0%) |  | 4 (1.5%) | 0 (0%) |  |
| Education years (IQR) |  | 9 (9-12) | 12 (9-12) | 0.27 | 9 (9-12) | 11 (9-12.5) | 0.17 |
| NIHSS at admission (IQR) |  | 2 (1-3) | 2 (1-4) | 0.03 | 2 (1-3) | 2 (1-4) | 0.28 |
| NIHSS at discharge (IQR) |  | 1 (0-2) | 1 (0-2) | 0.001 | 0 (0-2) | 1 (0-2) | 0.13 |
| mRs at discharge (%) |  |  |  | 0.003 |  |  | ＜0.001 |
|  | 0-2 | 270 (86.0%) | 42 (73.7%) |  | 237 (89.1%) | 75 (71.4%) |  |
|  | 3-6 | 44 (14.0%) | 15 (26.3%) |  | 29 (10.9%) | 30 (28.6%) |  |
| Hemiplegia (%) |  | 69 (22.0%) | 23 (40.4%) | 0.003 | 55 (20.7%) | 37 (35.2%) | 0.003 |
| Pain (IQR) |  | 0 (0-0) | 0 (0-0) | 0.84 | 0 (0-0) | 0 (0-0) | 0.22 |
| OSCP classification (%) |  |  |  | 0.06 |  |  | 0.18 |
|  | TACI | 17 (5.4%) | 2 (3.5%) |  | 14 (5.3%) | 5 (4.8%) |  |
|  | PACI | 169 (53.8%) | 32 (56.1%) |  | 139 (52.3%) | 62 (59.0%) |  |
|  | POCI | 68 (21.7%) | 19 (33.3%) |  | 60 (22.6%) | 27 (25.7%) |  |
|  | LACI | 60 (19.1%) | 4 (7.0%) |  | 53 (19.9%) | 11 (10.5%) |  |
| TOAST classification (%) |  |  |  | 0.06 |  |  | 0.01 |
|  | LAA | 112 (35.7%) | 17 (29.8%) |  | 95 (35.7%) | 34 (32.4%) |  |
|  | CE | 16 (5.1%) | 3 (5.3%) |  | 14 (5.3%) | 5 (4.8%) |  |
|  | SAO | 132 (42.0%) | 34 (59.6%) |  | 107 (40.2%) | 59 (56.2%) |  |
|  | ODE | 30 (9.6%) | 3 (5.3%) |  | 31 (11.7%) | 2 (1.9%) |  |
|  | UDE | 24 (7.6%) | 0 (0%) |  | 19 (7.1%) | 5 (4.8%) |  |
| Comorbidities (%) |  |  |  |  |  |  |  |
| Hypertension |  | 230 (73.2%) | 46 (80.7%) | 0.24 | 194 (72.9%) | 82 (78.1%) | 0.31 |
| Diabetes |  | 111 (35.4%) | 19 (33.3%) | 0.77 | 89 (33.5%) | 41 (39.0%) | 0.31 |
| Dyslipidemia |  | 65 (20.7%) | 11 (19.3%) | 0.81 | 53 (19.9%) | 23 (21.9%) | 0.67 |
| Chronic obstructive pulmonary disease |  | 1 (0.3%) | 1 (1.8%) | 0.71 | 1 (0.4%) | 1 (1.0%) | 0.49 |
| Atherosclerosis |  | 13 (4.1%) | 2 (3.5%) | 1.00 | 12 (4.5%) | 3 (2.9%) | 0.66 |
| Atrial fibrillation |  | 15 (4.8%) | 2 (3.5%) | 0.94 | 11 (4.1%) | 6 (5.7%) | 0.51 |
| Hyperuricemia |  | 3 (1.0%) | 1 (1.8%) | 0.49 | 2 (0.8%) | 2 (1.9%) | 0.68 |
| Coronary heart disease |  | 23 (7.3%) | 5 (8.8%) | 0.70 | 16 (6.0%) | 12 (11.4%) | 0.08 |
| BMI (IQR) |  | 24.5 (22.7-27.0) | 24.2 (22.9-26.1) | 0.15 | 24.4 (22.4-26.7) | 24.7 (23.35-26.9) | 0.52 |
| Prior stroke (%) |  | 89 (28.3%) | 11 (19.3%) | 0.16 | 81 (30.5%) | 19 (18.1%) | 0.02 |
| Carotid atherosclerosis (%) |  | 233 (74.2%) | 45 (78.9%) | 0.45 | 199 (74.8%) | 79 (75.2%) | 0.93 |
| Smoking (%) |  | 111 (35.4%) | 26 (45.6%) | 0.14 | 96 (36.1%) | 41 (39.0%) | 0.60 |
| Cognitive function (IQR) |  | 23 (19-27) | 21 (14.5-26) | 0.002 | 26 (21-28) | 21 (14.5-26) | 0.05 |
| Treatment methods for ischemic stroke (%) |  |  |  | 0.65 |  |  | 0.03 |
|  | Non-guideline recommended treatment | 256 (81.5%) | 45 (78.9%) |  | 226 (85.0%) | 75 (71.4%) |  |
|  | Guideline recommended treatment^a^ | 58 (18.5%) | 12 (21.1%) |  | 40 (15.0%) | 30 (28.6%) |  |
| Cognitive reserve (IQR) |  | 86 (80-98) | 87 (81-95.5) | 0.23 | 86.5 (80-99) | 85 (80-93) | 1.00 |

Abbreviations: BMI, Body mass index; IQR, interquartile range; mRs, Modified Rankin Scale; NIHSS, National Institutes of Health Stroke Scale; OCSP, Oxfordshire Community Stroke Project;TOAST, Trial of ORG 10172 in Acute Stroke Treatment
